# Financial IncEntives for Smoking TreAtment: protocol of the FIESTA trial and FIESTA Oral Microbiome Substudy

**DOI:** 10.1186/s13063-018-3003-y

**Published:** 2018-11-21

**Authors:** Katherine M. French, Sasha Z. Gonzalez, Scott E. Sherman, Alissa R. Link, Sadozai Zoe Malik, Chi-Hong Tseng, Saahil A. Jumkhawala, Briesny Tejada, Andrew White, Joseph A. Ladapo

**Affiliations:** 1 Department of Population Health, New York University School of Medicine, 227 East 30th Street, New York, NY 10016 USA; 2 Department of Medicine, VA New York Harbor Healthcare System, 423 East 23rd Street, New York, NY 10010 USA; 3 Department of Medicine, New York University School of Medicine, 560 First Avenue, New York, NY 10016 USA; 4 Division of General Internal Medicine and Health Services Research, David Geffen School of Medicine at UCLA, 10833 Le Conte Avenue, Los Angeles, CA 90095 USA; 5 Tufts University School of Medicine, 145 Harrison Avenue, Boston, MA 02111 USA; 6 New York Medical College, 40 Sunshine Cottage Road, Valhalla, NY 10595 USA; 7 SUNY Downstate Medical Center College of Medicine, 450 Clarkson Ave, Brooklyn, NY 11203 USA; 8 Division of General Internal Medicine and Health Services Research, David Geffen School of Medicine at UCLA, 911 Broxton Ave., Los Angeles, CA 90024 USA

**Keywords:** FIESTA, Smoking cessation, Manhattan VA Hospital, Financial incentives, Veterans

## Abstract

**Background:**

Smoking is the leading preventable cause of death in the United States, but evidence-based smoking cessation therapy is underutilized. Financial incentive strategies represent an innovative approach for increasing the use of counseling and pharmacotherapy. If effective, they could supplement or supplant resource-intensive policy options, particularly in populations for whom smoking has substantial societal costs. FIESTA (Financial IncEntives for Smoking TreAtment) will randomize hospitalized smokers to receive usual smoking cessation care alone or usual smoking care augmented with financial incentives. We aim to compare the impact of these two strategies on 1) smoking abstinence, 2) use of counseling and nicotine replacement therapy, and 3) quality of life of participants. We also will evaluate the short-term and long-term return on the investment of incentives. The FIESTA Oral Microbiome Substudy will compare the oral microbiome of smokers and nonsmokers to longitudinally assess whether smoking cessation changes oral microbiome composition.

**Methods:**

We will enroll 182 inpatient participants from the Manhattan campus of the Veterans Affairs New York Harbor Healthcare System. All participants receive enhanced usual care, including screening for tobacco use, counseling while hospitalized, access to nicotine replacement therapy, and referral to a state Quitline. Patients in the financial incentive arm receive enhanced usual care and up to $550 for participating in the New York Smoker’s Quitline, using nicotine replacement therapy (NRT), and achieving biochemically confirmed smoking cessation at 2 months and 6 months. In the microbiome substudy, we enroll nonsmoking control participants matched to each recruited smoker’s hospital ward, sex, age, diabetes status, and antibiotic use. After discharge, participants are asked to complete periodic phone interviews at 2 weeks, 2 months, 6 months, and 12 months and provide expired carbon monoxide and saliva samples at 2 months, 6 months, and 12 months for cotinine testing and oral microbiome analysis.

**Discussion:**

The incentive interventions of FIESTA may benefit hospitalized smokers, an objective made all the more critical because smoking rates among hospitalized patients are higher than those in the general population. Moreover, the focus of FIESTA on evidence-based therapy and bioconfirmed smoking cessation can help guide policy efforts to reduce smoking-related healthcare costs in populations with high rates of tobacco use and costly illnesses.

**Trial registration:**

ClinicalTrials.gov, NCT02506829. Registered on 1 July 2014.

**Electronic supplementary material:**

The online version of this article (10.1186/s13063-018-3003-y) contains supplementary material, which is available to authorized users.

## Background

Smoking is the leading preventable cause of death and disease in the United States [1], and most adults who continue to smoke are disproportionately low-income or have no college education [2]. While 70% of smokers express the desire to quit [3], only 25% of those individuals seek assistance with smoking cessation and an even smaller proportion use evidence-based methods [4], such as behavioral counseling or pharmacotherapy [5]. Among hospitalized patients, tobacco dependence treatment is particularly critical because persistent smoking increases the risk of future smoking-related illnesses, such as coronary heart disease, cerebrovascular disease, emphysema, and cancer [1, 6].

Financial incentives for smoking cessation may be an effective strategy for increasing the use of evidence-based cessation therapy among hospitalized patients [7–10]. Based on principles from microeconomic theory, financial incentives influence individual behavior and decision-making because individuals are motivated by the prospect of welfare gains. While evidence is mixed, some incentive interventions have been effective in the treatment of obesity [11], diabetes [12], and smoking at the worksite [9]. Incentives may be particularly effective when designed to leverage concepts from behavioral economics, such as immediacy of payments and framing of missed payments with regret aversion [7, 8, 13–17]. If effective, financial incentive programs could supplement or supplant other policy options for smoking cessation, particularly in populations for whom smoking has substantial societal costs. In addition, because health insurers are increasingly adopting bundled payment systems and smokers generally incur high healthcare costs [8, 13, 18], financial incentives may be sustainable due to their potentially favorable return on investment [14, 15]. The effectiveness of financial incentives for increasing smoking cessation among hospitalized patients is unknown.

In addition to the potential effectiveness of incentives for smoking cessation, another scientific area of growing relevance to smokers is the oral microbiome. Smokers have higher levels of oral mutagens and carcinogens, and this may contribute to their increased risk of oral cancer. It is also known that quitting smoking reduces the risk of developing oral cancer [19]. Recent research has suggested that a mechanism explaining the association of smoking and oral cancer may involve activation of carcinogens deposited through smoking by oral bacteria [20]. However, little is known about how smoking cessation affects the oral microbiome of smokers.

### Objectives

The primary objective of the Financial IncEntives for Smoking TreAtment (FIESTA) trial is to compare the effects of two approaches for smoking cessation—financial incentives plus enhanced usual care versus enhanced usual care alone—on smoking abstinence, use of evidenced-based therapy, and quality of life. The second objective is to estimate the short-term and long-term return on investment of using financial incentives to promote smoking cessation. In addition, the FIESTA Oral Microbiome Substudy will be conducted to determine whether smoking cessation changes the oral microbiome composition of smokers.

We hypothesize that participants in the financial incentives arm will experience higher rates of smoking cessation, improved quality of life, and lower rates of financial distress, and that these gains will be associated with a favorable return on investment. We also hypothesize that patients experiencing greater financial distress at baseline will be more likely to stop smoking in response to financial incentives. Our oral microbiome hypothesis is that smoking cessation will lead to changes in the microbiome that increase its similarity to the microbiome of nonsmokers.

## Methods/design

### Overall design

**Fig. 1:**
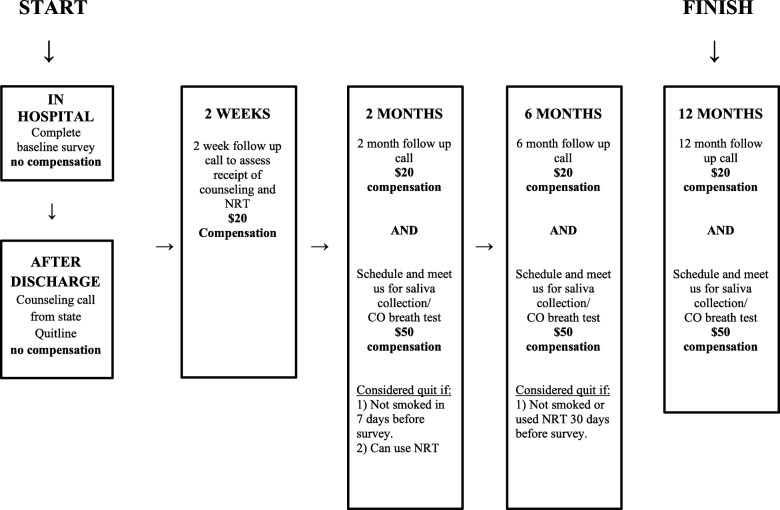
Brochure provided to participants in enhanced usual care arm summarizing time points for follow-up and compensation. NRT nicotine replacement therapy, VA Veteran’s Affairs

**Fig. 2:**
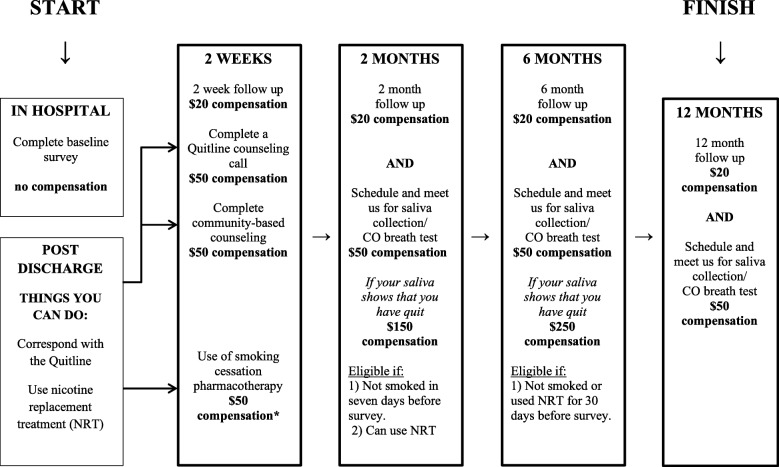
Brochure provided to participants in financial incentives arm summarizing time points for follow-up and compensation. NRT nicotine replacement therapy, VA Veteran’s Affairs

The trial is sponsored by the Robert Wood Johnson Foundation. We will enroll 182 inpatient participants from the Manhattan campus of the Veterans Affairs (VA) New York Harbor Healthcare System. After conducting a baseline survey of the sociodemographic and clinical characteristics of participants and administering a Stages of Change questionnaire, smokers are randomized to the financial incentives plus enhanced usual care arm or the enhanced usual care alone arm with an allocation ratio of 1:1. All participants receive enhanced usual care, which includes tobacco use screening, counseling while hospitalized, hospital-directed educational materials on quitting smoking, nicotine replacement therapy (NRT) at the discretion of inpatient healthcare providers, and referral to a state Quitline (this component represents the enhancement). The components of enhanced usual care were selected based on prior evidence for effective smoking cessation interventions. In addition to enhanced usual care, smokers who are randomized to the financial incentives arm are also eligible to receive up to $550 for participating in counseling (both community-based counseling and state Quitline counseling), using smoking cessation pharmacotherapy, and biochemically confirmed smoking cessation at 2 months and 6 months. The incentive schedule is summarized in Table 1, and Figs. 1 and 2 display brochures provided to participants in the enhanced usual care arm or the financial incentives plus enhanced usual care arm.

**Table 1:** Schedule of financial incentives

Activity	Time point	Incentive
Speaking with a coach from the NYS Smoker’s Quitline	2 weeks	$50
Completion of community-based smoking cessation program	2 weeks	$50
Use of pharmacotherapy for smoking cessation	2 weeks	$50
Smoking cessation (self-report + salivary cotinine)	2 months	$150
Smoking cessation (self-report + salivary cotinine)	6 months	$250

**Fig. 3:**
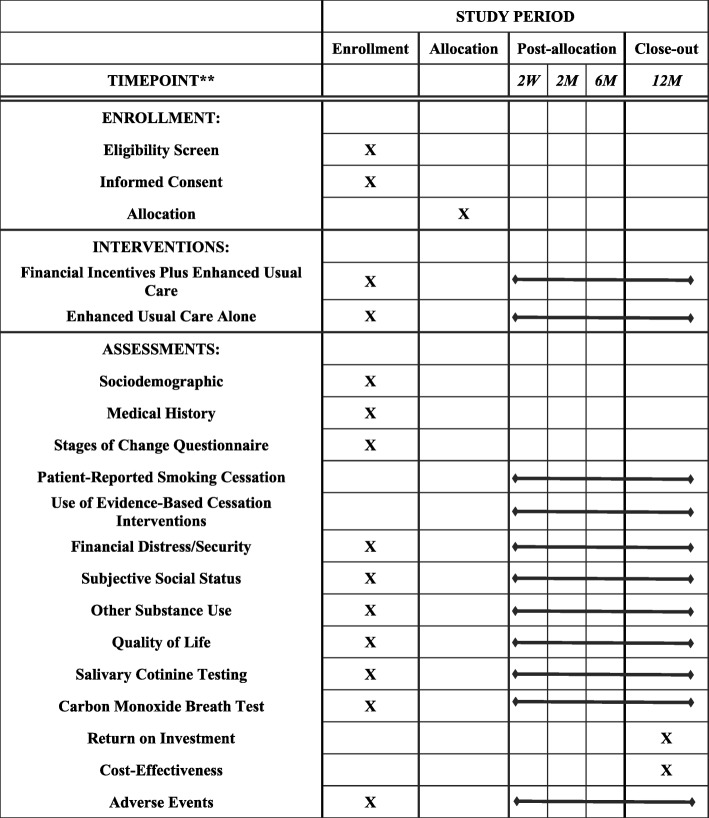
Schedule for enrollment, interventions, and assessments (SPIRIT figure)

After discharge, smokers are asked to complete periodic phone interviews for 12 months to assess patient-reported smoking cessation, use of evidence-based cessation interventions, and quality of life. All participants are also asked to provide saliva samples for 12 months for both cotinine testing and oral microbiome analysis. Smoking status at 6 months, as determined by salivary cotinine analysis, is used to determine whether a patient is considered a persistent smoker or a past smoker because rates of recidivism are low after 6 months [21], while many patients who quit smoking immediately after being hospitalized soon relapse [16]. An outline of enrollment, intervention, and assessment can be found in the SPIRIT figure (Fig. 3) and SPIRIT checklist (Additional file 1).

### Study population

The VA Hospital in Manhattan is a New York University (NYU) School of Medicine-affiliated teaching facility. The institution predominately serves low-income veterans that reside in Manhattan or its surrounding regions. The low socioeconomic status of most participants in this trial may limit the generalizability of our findings to other socioeconomic groups within the United States and elsewhere. Each year, approximately 4000 patients are admitted to its medical and surgical services, while another approximately 1000 patients are admitted to its psychiatry service. Roughly 60% of hospitalized patients at the Manhattan VA are Caucasian, 31% are African American, and 17% are Hispanic or Latino. Furthermore, about 61% of these patients have only a high school education or less. The institution routinely cares for patients with acute myocardial infarction, congestive heart failure, pneumonia, and chronic obstructive pulmonary disease—all conditions associated with smoking.

### Eligibility criteria

The criteria for hospitalized patients considered for enrollment in FIESTA are: 1) ≥ 18 years of age; 2) smoked tobacco during the 30 days prior to hospitalization; 3) possess an active US phone number; 5) not pregnant or breastfeeding; 6) reside in the NYC area or have the ability to return to the Manhattan VA for at least 1 year; 7) contemplating smoking cessation as assessed by readiness to quit [22]; and 8) able to provide consent in English. Potential participants are excluded if they: 1) use smokeless tobacco only; 2) are pregnant or breastfeeding; 3) anticipate to be discharged to an institution (i.e., a nursing home or long-term care facility) at which the patient will not have control over smoking rules; or 4) are unable to provide informed consent.

### Recruitment

The Veterans Information Systems and Technology Architecture (VISTA) program is used to gather information from the Electronic Health Record (EHR) system (specifically from nursing or physician admission assessments) to identify hospitalized smokers who are eligible for participation. Each day, an EHR-generated current smokers list is created and used to recruit. Staff approach every potential participant to describe the study, assess eligibility through the completion of a screener, offer enrollment if eligible, complete a Research Consent Form and HIPAA Release Form, and conduct a baseline survey and saliva collection. All electronic data are kept in a password-protected and encrypted database (REDCap) and each participant is assigned a study ID number to separate their identifiable information from the study records. Potential participants are also informed that their participation is completely voluntary, and their consent can be withdrawn at any time by providing staff with a letter requesting they no longer be contacted.

The FIESTA Oral Microbiome Substudy will enroll both smokers and hospitalized nonsmokers as controls. For each smoker enrolled in FIESTA, staff will attempt to recruit a matched nonsmoker using VISTA and electronic chart review. Nonsmokers are matched based on: 1) age within 10 years (if a match is identified in the first 30 days after enrollment of the corresponding FIESTA participant) or within 20 years (after the first 30 days); 2) sex; 3) ward location; 4) antibiotic use; and 5) diabetes status. We will relax the matching criterion based on diabetic status if we are otherwise unable to identify a match.

The Computerized Patient Record System (CPRS) is used to track the discharge information of participants and to complete a chart abstraction 1 week postdischarge. After discharge, smokers are referred to a state Quitline (either New York or New Jersey) by research staff and later contacted by a state Quitline counselor to discuss NRT, plans and strategies for quitting, and other available smoking cessation resources.

### Randomization

Smokers are randomized in a 1:1 ratio to either financial incentives plus enhanced usual care or enhanced usual care alone. We employ a computer-generated block randomization design. Research staff implement the allocation sequence using numbered, sealed envelopes that are opened during participant enrollment. Because of the nature of the intervention, participants are not blind to their randomization into either the financial incentives plus enhanced usual care or enhanced usual care groups.

### Intervention

Smokers who are randomized into the financial incentives plus enhanced usual care arm are eligible to receive $550 for participating in counseling, using NRT, and achieving biochemically confirmed smoking cessation at 2 months and 6 months (see Table 1). All participants are compensated in US dollars (USD) using ClinCards, a secure prepaid debit card system.

Research staff members are extensively trained in the teach-back method in which study participants are asked to repeat task-specific directions to staff to confirm understanding. The teach-back method has been shown to improve instruction retention in patients postdischarge [23]. This instructional technique will be incorporated into the FIESTA protocol to ensure that participants understand not only the study aims and timeline, but have also comprehended the targets for which they are being incentivized.

### Data collection and measurement of smoking cessation outcomes

Participants are asked to complete an in-person survey at enrollment and four follow-up interviews by phone at 2 weeks, 2 months, 6 months, and 12 months. Each participant receives $20 after completing surveys at each of the follow-up time points. Each participant is also asked to provide saliva samples at enrollment and at 2 months, 6 months, and 12 months, for which we provide a $50 payment to increase participation rates. The baseline and follow-up surveys are similar in content and assess: 1) sociodemographic characteristics (i.e., demographics, contact information, race/ethnicity, health literacy, exercise habits, and nutrition habits) [24]; 2) smoking history (i.e., smoking habits and home environment using questions adapted from the California Tobacco Survey [25], nicotine dependence using questions adapted from the Heaviness of Smoking Index [26], and smoking cessation services received); 3) preference for incentive design (goal-directed versus outcome-based); 4) participant health (i.e., health status using the Veterans RAND 12-item Health Survey [27] and the EuroQol-5D [28–30] to assess physical and mental functioning, alcohol-use using the AUDIT-C [31, 32], marijuana use using the ASSIST, substance use using the self-administered Substance Use Brief Screen [33], and depression using the PHQ-2 [34]); and 5) health services utilization in the prior 6 months. The baseline and follow-up surveys also measure treatment outcomes (i.e., 7-day and 30-day point prevalence for smoking cessation, quit attempts, reduction in daily smoking, changes in readiness to quit, and use of NRT and Quitline counseling).

We collect saliva samples from each participant at baseline, 2 months, 6 months, and 12 months. The sample is analyzed for cotinine—a biomarker that reflects exposure to nicotine—using NicAlert kits to bioconfirm smoking cessation, and the sample is also used to characterize the oral microbiome. All participants also undergo a carbon monoxide breath test at every saliva collection. In addition, we ask each participant if they have taken antibiotics in the 7 days prior to saliva collection or used NRT, e-cigarettes, or other forms of tobacco in the 7 days prior to saliva collection. At 2 months, if a participant reports tobacco abstinence, but is still using NRT and the cotinine testing results in a value between 10 and 99 ng/mL, we use the carbon monoxide breath test for biochemical validation. Research staff collect all saliva samples in public locations convenient to the patient or at the Manhattan VA.

Research coordinators periodically audit study procedures every 3 to 6 months to ensure implementation fidelity and adherence to the study protocol. Routine re-training of research staff to reinforce study procedures is performed every 6 to 9 months. This process is performed independently of the investigators, but impressions of study staff performance and feedback from re-training exercises are shared with the principal investigators.

### Incentive program preferences

To assess preference for incentive design, research staff describe two hypothetical financial incentive programs for smoking cessation, and then ask which program the participant prefers. One program uses goal-directed incentives (incentives weighted toward use of evidence-based therapies) and the other uses outcome-based incentives (incentives for successful achievement of an outcome such as successfully quitting) [5]. Total attainable incentives are similar in both programs. These data inform hypotheses regarding whether patients randomized to the incentive arm will demonstrate different smoking cessation outcomes depending on their prespecified (prerandomization) program preference.

### Oral Microbiome Substudy

The FIESTA Oral Microbiome Substudy aims to assess whether smoking cessation changes oral microbiome composition by comparing the oral microbiome of smokers and nonsmokers longitudinally. The FIESTA Oral Microbiome Substudy has four objectives. The first objective is to investigate differences in the oral microbiomes of smokers versus nonsmokers at baseline. We hypothesize that there will be significant, dose-related differences. The second objective is to determine if smoking is associated with changes in the oral microbiome longitudinally. We hypothesize that the oral microbiomes of quitters will be most similar to nonsmokers at 12 months. The third objective is to identify oral microbiome predictors of successful smoking cessation by comparing the baseline oral microbiome of smokers who successfully quit to the baseline oral microbiome of smokers who did not quit. We hypothesize that there will be no specific patterns that are predictive of success in smoking cessation. The fourth objective is to determine if the oral microbiome in smokers is associated with increased levels of oral mutagens and carcinogens. We hypothesize that smokers will have higher baseline levels of mutagens and carcinogens and that quitters will have levels of mutagens and carcinogens comparable to nonsmokers after 12 months.

To achieve the objectives of the FIESTA Oral Microbiome Substudy, we enroll nonsmoking control participants matched to each FIESTA smoker based on the criteria previously described. Saliva samples are collected at baseline, 2 months, 6 months, and 12 months to characterize the oral microbiome. Specimens are stored in a freezer at approximately −78 °C in the Manhattan VA until laboratory analysis is conducted. We complete an Ames test and test for the carcinogens benzo-a-pyrine and nicotine-derived nitrosamine ketone. This is a pilot performed on a subset of participants. We also assess how the oral flora metabolized carcinogens.

For data collection, each participant is asked to complete an in-person survey at enrollment in addition to follow-up interviews by phone at 2 months, 6 months, and 12 months. These participants are also asked to provide saliva samples at these time points, with a $50 incentive payment at each follow-up time point. Letters are mailed to participants 2 weeks before each follow-up window as a reminder and to provide them with the opportunity to update their contact information.

### Adverse events

At all scheduled contact between staff and participants, patients are asked about any potential adverse events that had occurred since enrollment or their most recent follow-up assessment. This list of potential events includes violations of confidentiality, emergency room visits without hospitalization, and suicidal ideation not requiring intervention, as well as serious adverse events, including death, life-threatening events, hospitalization, and suicidal ideation requiring intervention. If a patient experiences an adverse event, staff record: 1) event onset date; 2) report date; 3) reporting staff member; 4) study phase, i.e., prerandomization, in-treatment, or follow-up; and 5) a detailed description of the adverse event, i.e., symptoms, course, duration, treatment, or cause of death if known.

### Data monitoring

The study coordinators generate reports for the principal investigators on a monthly basis. These reports contain a summary of enrollment, retention, participant withdrawals, detailed descriptions of adverse events, and detailed descriptions of protocol violations. Reports are periodically submitted to the Institutional Review Board (IRB) for continued review. The principal investigators are notified within 24 h of recognition of any adverse events. Serious adverse events are reported within 10 business days to the IRB.

### Sample size

We will enroll 182 smokers and expect a 60% participation rate. In addition, we expect a 10–15% loss to follow-up rate at 6 months. We consider an absolute difference of approximately 20% in smoking cessation rates to be clinically substantial, where absolute difference = (rate_1_ – rate_2_). Therefore, this sample size (91 patients per arm) allows at least 80% power to detect a substantial clinical difference in smoking cessation rates between the control group (enhanced usual care alone arm) and intervention group (financial incentives plus enhanced usual care arm) with α = 0.05.

### Analytic plan

The primary outcome in this trial is the smoking cessation rate at 6 months. Secondary outcomes in this trial include: 1) financial distress and security; 2) subjective social status; 3) other substance use including alcohol; and 4) quality of life. Chi-squared tests, *t* tests, and nonparametric tests will be used to compare baseline characteristics of patients to assess balance between the two study arms. Multivariate analyses for longitudinal data will include difference-in-difference models and Cox proportional hazard models, and these models will include patient-level variables, area-based socioeconomic factors, and hospital variables as covariates.

For the economic evaluation, we will estimate the return on investment of financial incentives intervention from the perspective of the healthcare system (hospitalizations, ambulatory care, and medications) on a per-patient basis. Costs will be determined by: 1) multiplying employee wages (based on United States Bureau of Labor Statistics values) [35] by the estimated time these personnel spend on smoking cessation care; 2) using the Red Book to estimate NRT and other medication costs (based on average wholesale prices) [36]; and 3) estimating bulk purchase prices for other physical materials provided to smokers. The return on investment will then be estimated using the difference between the value of financial incentives provided to smokers and the incremental healthcare costs or savings, comparing the financial incentives plus enhanced usual care arm to the enhanced usual care alone arm.

We will also estimate the cost-effectiveness of the financial incentives intervention using the ratio of the difference in costs between the intervention group and control group to the difference in quality of life and smoking cessation rates between the intervention and control groups [37]. We will perform nonparametric bootstrapping with 1000 random samples to estimate confidence intervals (CIs) for the cost-effectiveness ratios and we will use the bias-corrected percentile method described by Efron and others [38–40]. We will consider recidivism rates of participants when estimating the cost-effectiveness of the interventions in this trial. Specifically, we anticipate that recidivism will reduce cost-effectiveness by reducing the incremental benefits of the intervention.

## Discussion

### Innovation

FIESTA is innovative for three main reasons. FIESTA is the first randomized trial to test the effectiveness of financial incentives in hospitalized patients. Previous smoking cessation studies enrolling inpatient smokers have utilized different intervention models, with follow-up counseling after discharge being one of the most commonly used models. In a 2012 meta-analysis of smoking cessation studies, the authors found that supportive follow-up counseling for at least 1 month postdischarge—provided by either nurses or trained counselors—significantly increased smoking cessation rates [41]. Other studies in this meta-analysis concluded that adding NRT to intensive counseling increased smoking cessation rates compared with intensive counseling alone (risk ratio (RR) 1.54, 95% CI 1.34 to 1.79, six trials). Because this meta-analysis largely examined interventions incorporating intensive counseling, its results may not be generalizable to our study, which leverages a more pragmatic model [41]. While FIESTA does not formally incorporate 4 weeks of postdischarge follow-up per se, it leverages a pragmatic framework by building on Quitlines, an existing infrastructure for evidence-based smoking cessation.

A second innovative characteristic of FIESTA is its formal incorporation of an economic analysis to evaluate return on investment. At the time of FIESTA’s design, economic evaluations were uncommon in smoking cessation trials of hospitalized patients. In FIESTA, this economic evaluation explicitly addresses the issue of financial sustainability of financial incentives for smoking cessation.

A third innovative component of FIESTA is its incorporation of behavioral economic concepts. While limited in scope when considering the expansive number of tools available in the behavioral economic literature, FIESTA emphasizes the importance of providing payments as soon as possible, so that participants could more readily associate a payment with the behavior that triggered it. Behavioral economists often cite hyperbolic discounting as an explanation for why money received immediately is perceived to be substantially more valuable than money received in the near future, even after accounting for standard discounting. We also frame feedback to participants in the incentive arm who did not meet their goals using regret aversion by emphasizing what the participants would have received had they met their goals [42, 43].

FIESTA also faces several challenges. Our patient population consists largely of low-income participants and they frequently face socioeconomic burdens (housing instability, substance abuse, etc.) that increase the challenges associated with recruitment and retention. With regard to recruitment, research staff often encounter barriers to recruitment that are inherent to the hospital setting, such as surgical operations or procedures, nurse or physician visits, family or friend visits, meals, and uncertainty about postdischarge disposition. Consequently, it is frequently difficult to screen and enroll eligible patients prior to discharge. Moreover, in some instances, the postdischarge disposition of participants changes following enrollment, and the participants will instead enter a rehabilitation program during which we are unable to reach them. In addition, many participants use prepaid cell phone plans that are restricted in the number of available minutes per month, which likely reduces the likelihood of successfully completing follow-up surveys or scheduling saliva collection appointments with research staff. Many participants also lack permanent home addresses and often change addresses depending on housing or shelter availability, which likely decreases the probability that participation letters or mailed reminders will reach the intended participant.

### Summary and significance

FIESTA is the first randomized trial of financial incentives for smoking cessation in hospitalized patients, and it focuses on hospitalized veterans. We anticipate that its findings will have implications for our understanding of the effectiveness of financial incentive interventions in inpatient settings, the association of smoking cessation with oral microbiome changes, and the design of smoking cessation interventions in vulnerable hospitalized populations.

### Trial status

FIESTA began enrollment on 14 July 2015 and enrollment is ongoing. The protocol version used was Version 1, dated 19 March 2015.

## Additional file

Additional file 1: SPIRIT checklist. (DOC 125 kb)

## Abbreviations

EHR: Electronic Health Record
FIESTA: Financial IncEntives for Smoking TreAtment
NRT: Nicotine replacement therapy
VA: Veteran’s Affairs
VISTA: Veterans Information Systems and Technology Architecture
